# GBAS Ionospheric Anomaly Monitoring Based on a Two-Step Approach

**DOI:** 10.3390/s16060763

**Published:** 2016-05-26

**Authors:** Lin Zhao, Fuxin Yang, Liang Li, Jicheng Ding, Yuxin Zhao

**Affiliations:** 1 College of Automation, Harbin Engineering University, Harbin 150001, China; zhaolin@hrbeu.edu.cn (L.Z.); yangfuxin@hrbeu.edu.cn (F.Y.); aaron.heu@163.com (J.D.); zhaoyuxin@hrbeu.edu.cn (Y.Z.); 2 Academy of Opto-Electronics, Chinese Academy of Sciences, Beijing 100094, China

**Keywords:** GPS receiver, ionospheric gradient anomaly, CCD, two-step approach (TSA), response time, estimation accuracy, detection sensitivity

## Abstract

As one significant component of space environmental weather, the ionosphere has to be monitored using Global Positioning System (GPS) receivers for the Ground-Based Augmentation System (GBAS). This is because an ionospheric anomaly can pose a potential threat for GBAS to support safety-critical services. The traditional code-carrier divergence (CCD) methods, which have been widely used to detect the variants of the ionospheric gradient for GBAS, adopt a linear time-invariant low-pass filter to suppress the effect of high frequency noise on the detection of the ionospheric anomaly. However, there is a counterbalance between response time and estimation accuracy due to the fixed time constants. In order to release the limitation, a two-step approach (TSA) is proposed by integrating the cascaded linear time-invariant low-pass filters with the adaptive Kalman filter to detect the ionospheric gradient anomaly. The performance of the proposed method is tested by using simulated and real-world data, respectively. The simulation results show that the TSA can detect ionospheric gradient anomalies quickly, even when the noise is severer. Compared to the traditional CCD methods, the experiments from real-world GPS data indicate that the average estimation accuracy of the ionospheric gradient improves by more than 31.3%, and the average response time to the ionospheric gradient at a rate of 0.018 m/s improves by more than 59.3%, which demonstrates the ability of TSA to detect a small ionospheric gradient more rapidly.

## 1. Introduction

As one significant component of space environmental weather, the ionosphere is one of the critical error sources for a diversity of applications using satellite navigation, e.g., farming, construction, exploration, surveying and civil aviation [1]. Although, the effect of the ionosphere on the Global Navigation Satellite System (GNSS) could be weakened by modeling or dual-frequency combination using the GNSS receiver, which is the key sensor for satellite navigation [2,3,4]. The ionosphere also has to be monitored; this is because the ionospheric anomaly, which cannot be detected in a timely manner, would yield a false position result or even a disaster outcome for safety-of-life applications [5]. For example, the precision aircraft landing supported by the Ground-Based Augmentation System (GBAS) is vulnerable to rare ionospheric anomalies [6]. Since the estimation accuracy and response time of ionospheric anomaly monitoring are very demanding and challenging for GBAS relative to other safety-critical services [7,8,9], therefore, how to detect an ionospheric anomaly rapidly and accurately is the potential research direction of ionospheric anomaly monitoring for GBAS.

GBAS is implemented by the technique of code-based differential positioning, which aims at improving the positioning accuracy and reliability simultaneously by broadcasting the differential corrections and integrity information from a reference station. The typical implementation of GBAS is the Local Area Augmentation System (LAAS), which has been widely used in civil aviation to aid the precision of the approach and landing. Aiming at improving positioning accuracy, LAAS has recommended the utilization of the carrier-smoothed-code (CSC) technique to suppress the error of the multipath and receiver noise in code measurements.

Besides the single-frequency-based combination, the CSC can also be categorized into another two groups with respect to the combination of dual-frequency code and phase observations, *i.e.*, the divergence-free (DFree) model and the ionosphere-free (IFree) model [10,11,12,13]. However, the DFree and IFree models are not yet available for civil aviation applications, as the second GPS frequency (L2) does not fall in a protected Aeronautical Radio Navigation Service (ARNS) band and the proposed third GPS frequency (L5) is not widely available currently [14]. Meanwhile, the cost of the dual-frequency receiver is too high to be widely used. Therefore, the single-frequency Hatch filter model has been recommended by GBAS.

Although the positioning accuracy is improved by the single-frequency smoothed pseudorange in normal conditions, the ionospheric gradient, which is a dominant threat for GBAS during ionosphere storm events, will be introduced into the smoothed pseudorange corrections due to the fact that the ionosphere affects satellite signal propagation by lagging code measurements while leading carrier-phase measurements, namely code-carrier divergence (CCD) [5]. Many ionospheric gradient anomaly events, which are mainly caused by solar storms, have been observed, for example in 2000 and 2003 [15]. It has been shown that differential ranging errors due to the abnormal ionospheric gradient between the reference and users could exceed 3–5 m in a baseline of less than 5 km during ionosphere storm hits [16]. Therefore, the ionosphere storm events can create unacceptable positioning error, even over a short baseline, which could be a disaster for safety-of-life applications.

**Figure 1:**
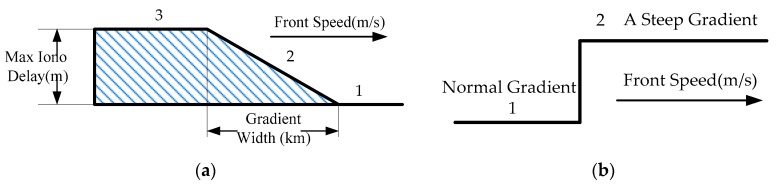
(**a**) Simplified model of the ionosphere anomaly; (**b**) simplified model of the ionosphere gradient anomaly.

For GBAS, the ionospheric anomaly model can always be modeled as a steep traveling wave-front between regions of low (*i.e.*, 3) and high (*i.e.*, 1) ionospheric delay depending on a large amount of GPS data, as shown in Figure 1 [15]. The wave-front is described as a piecewise linear curve by three parameters: max ionospheric delay, front speed and gradient width. Figure 1a shows a linear change in 2 between 1 and 3. The steady accumulated error of single frequency carrier smoothing will be much larger if a steep gradient in the ionospheric is delayed; in this case, a huge degradation of accuracy would happen [17]. Therefore, we focus on the ionospheric gradient anomaly, which is a dominant threat for GBAS, rather than the ionospheric delays’ anomaly. The ionospheric gradient anomaly model in Figure 1b can be derived from Figure 1a.

In order to ensure the integrity of code-based differential positioning by the CSC technique in GBAS, many researchers are working on improving the estimation accuracy of the ionospheric gradient and response time to the ionospheric gradient anomaly simultaneously, namely CCD monitoring. For the purpose of meeting the Minimal Operational Performance Standards (MOPS) requirements on the ionospheric gradient anomaly for LAAS [18,19], a geometric moving averaging (GMA) method of a linear time-invariant first-order low-pass filter (called CCD-1OF) is used to consider both the estimate accuracy of the ionospheric gradient and the response time to the ionospheric gradient anomaly, as the recommended integrity monitoring algorithm of LAAS [20]. However, there is a counterbalance that the accuracy of estimation and the response time to the anomaly cannot be improved simultaneously with a fixed time constant. Then, Kim designed a feasible generalized least square (GLS) method to estimate the ionospheric gradient, assuming that the gradient is a constant over tens of minutes [21]. Nevertheless, the estimation accuracy is barely satisfactory, because the integer ambiguity solution cannot be fixed, and the response time is too long to detect the ionospheric gradient. In order to obtain real-time ionosphere delay, Ouzeau suggested a traditional Kalman filter method to estimate both ionosphere delay and integer ambiguity [22]. Although this method satisfies the real-time requirement, the estimation accuracy will be affected when the system model and the actual situation are mismatch. In addition, the line-of-sight ionosphere delay can also be estimated accurately by precise point positioning; however, being in real time cannot be guaranteed, and the estimation accuracy is easily affected by abnormal satellite attitude [23,24]. On the basis of CCD-1OF, Simili has proposed a cascaded first-order linear time-invariant low-pass filter method (called CCD-2OF) to improve both the response time to the anomaly and the estimation accuracy [25]. However, there is a common shortcoming between CCD-1OF and CCD-2OF with a fixed time constant.

Considering the MOPS requirements for LAAS on estimation accuracy and response time, in order to release the limitation between the estimation accuracy and the response time by traditional CCD methods with a fixed time constant, a TSA is introduced by integrating a cascaded first-order linear time-invariant low-pass filter with an adaptive Kalman filter based on the traditional GPS receiver. Firstly, the *a priori* information of the adaptive Kalman filter is calculated in the first step of TSA, and the real-time accuracy of the ionosphere increases by an adaptive Kalman filter for a non-stationary system in the second step, compared to the traditional CCD methods.

The remainder of paper is organized as follows. Firstly, the counterbalance between the response time and estimation accuracy is analyzed based on the MOPS requirements for LAAS, followed by a description of traditional CCD methods. Secondly, a TSA is proposed to monitor the first-order ionospheric gradient anomaly by integrating a cascaded first-order linear time-invariant low-pass filter with an adaptive Kalman filter. Thirdly, numerical and real GPS data simulations are provided to demonstrate the proposed approach, compared to the traditional methods. Finally, a conclusion and a discussion will follow.

## 2. Limitations of the Traditional CCD Methods

Both the CCD-1OF and CCD-2OF methods adopt a linear time-invariant low-pass method to estimate the ionospheric gradient by suppressing the high frequency noise. Nevertheless, both of them have a common limitation that the response time and estimation accuracy cannot be improved simultaneously with a fixed time constant. In order to analyze the limitation, the traditional CCD models are provided first.

### 2.1. Traditional CCD Methods

The code and carrier-phase measurements can be modeled as,

(1) $$\rho_{k}=r_{k}+\delta t_{s}+I_{k}+T_{k}+\delta t_{u}+m_{k}+\varepsilon_{\rho ,k}$$

(2) $$\varphi_{k}=r_{k}+\delta t_{s}-I_{k}+T_{k}-\lambda N_{k}+\delta t_{u}+\varepsilon_{\varphi ,k}$$

where *ρ* is the pseudo-range measurement between the receiver and satellite, *φ* is the carrier-phase measurement, *r* is the geometric range between the receiver and satellite, $\delta t_{s}$ and $\delta t_{u}$ are the satellite and receiver clock error, respectively, *I* and *T* are the ionospheric and tropospheric error, respectively, *N* is the integer ambiguity, λ is the carrier wavelength, *m* represents the pseudo-range multipath error, with the effect of multipath on the carrier phase being assumed to be zero, and $\varepsilon_{\rho }$ and $\varepsilon_{\varphi }$ are the pseudo-range and carrier-phase measurement errors, respectively. The subscript *k* indicates the *k*-th epoch.

From Equations (1) and (2), the code minus carrier (CMC) in the *k*-th epoch can be written as,

(3) $$z_{k}=2I_{k}+\lambda N+m_{k}+\varepsilon_{\rho ,k}-\varepsilon_{\varphi ,k}$$

Assuming the absence of cycle slips, the ionospheric gradient is introduced in the difference of the adjacent epoch CMC as follows,

(4) $$z_{k}-z_{k-1}\approx 2T_{s}I_{g,k}+m_{k}-m_{k-1}+\varepsilon_{\rho ,k}-\varepsilon_{\rho ,k-1}$$

where *T_s_* is the sample time and *I_g_* represents the ionospheric gradient. With the use of Equation (4), it can be found that the difference of the adjacent epoch CMC includes the double ionospheric gradient and multipath receiver noise. The noise greatly affects the estimation accuracy of the ionospheric gradient, when it is much bigger. Therefore, the traditional CCD methods normally use the linear time-invariant low-pass method to estimate the ionospheric gradient by suppressing the high frequency noise. The test statistic of CCD-1OF is expressed as [22],

(5) $$Div_{k}^{1}=\frac{\tau_{d1}-T_{s}}{\tau_{d1}}Div_{k-1}^{1}+\frac{1}{\tau_{d1}}\left(z_{k}-z_{k-1}\right)$$

where *τ_d_*_1_ is the time constant for the first-order linear time-invariant low-pass filter model, *T_s_* is the sample time and $Div_{k}^{1}$ is the ionospheric gradient estimation by attenuating the high-frequency component.

To expedite the detection of the ionospheric gradient anomaly, a CCD-2OF method is proposed by using two first-order linear time-invariant low-pass filters to suppress the high frequency noise to the test statistic by replacing Equation (5) with the following [25],

(6) $$Div_{k}^{2}=\frac{\tau_{d2}-T_{s}}{\tau_{d2}}Div_{k-1}^{2}+\frac{1}{\tau_{d2}}Div_{k}^{1}$$

where *τ_d_*_2_ is the time constant and $Div_{k}^{2}$ is the ionospheric gradient estimation by CCD-2OF. This method not only decreases the level of signal fluctuation with two relatively small time constants, but also increases the detection speed under the ionospheric gradient anomaly conditions, which meet the further MOPS requirements for LAAS on CCD monitoring [25].

After the test statistics of traditional CCD methods are chosen, in order to detect the ionospheric gradient anomaly, the detection threshold should be determined first. The detection threshold can be calculated based on the statistical parameter of the test statistics normally computed using the mean and the standard deviation of the estimator in terms of satellite elevations under fault-free conditions [11],

(7) $$T(el)=\mu (el)\pm K_{ffd}\cdot f\cdot \sigma (el)$$

where μ(el) and σ(el) are the mean and standard deviation of test statistics at the elevation angle of *el*, respectively. *K_ffd_* is a multiplier, so that the monitor can meet the false alarm requirement, *f* is an inflation factor to over-bound the heavy-tailed distribution of the test statistic. The detection threshold reflects the ability to detect the minimum change of the ionospheric gradient.

Although the CCD-2OF is superior to the CCD-1OF with their corresponding time constants on both estimation accuracy and response time to the anomaly, there is a limitation that the estimation accuracy and response time to the anomaly cannot be improved simultaneously [25]. This will be discussed in the time domain and the frequency domain, respectively.

### 2.2. Limitation between Estimation Accuracy and Response Time to Anomaly

The MOPS provides significant flexibility to avionics manufacturers by quantitatively specifying that the airborne filter need only match the ground filter with the following requirements for LAAS [25]:

“The nominal ionospheric CCD rate is given in The Local Area Augmentation System (LAAS) Ground Faclity (LGF) Specification as normally distributed with zero mean and standard deviation of 0.018 m/s. In response to a CCD rate of up to 0.018 m/s, the smoothing filter output shall achieve an error less than 0.25 m within 200 s after initialization relative to the steady-state response of filters specified in LGF.”

Although the MOPS requirements above are aimed at CSC, the ionospheric gradient, which is a dominant threat for GBAS during ionosphere storm events, will be introduced into the CSC due to the CCD effect. In order to ensure the integrity for the CSC, especially for the ionospheric gradient anomaly, the CCD monitoring method must be designed based on the requirements for the CSC. Therefore, the MOPS above for the CSC can equivalently be the threshold of the ionospheric gradient for CCD. From the MOPS requirements for LAAS on the CSC above, we know that the estimation accuracy of the ionospheric gradient and response time to the ionospheric gradient anomaly must be considered simultaneously, when a CCD monitoring model is designed.

Firstly, in order to meet the MOPS on response time to anomaly and the steady error, the relationship between the response time, the steady error and the time constant are discussed for CCD-1OF, respectively. From Equation (4), when z_k_ − z_k−1_ is regarded as the unit ramp input, the transfer function Φ(*s*) in the S-domain is expressed as,

(8) $$\Phi (s)=\frac{1}{\tau_{d1}\cdot (s+a)}$$

where a is in inverse proportion of the time constant and can be written as,

(9) $$a=\frac{\ln(1+\frac{1}{\tau_{d1}-1})}{T_{s}}$$

Assuming that the sample time *T_s_* is 1 *s*, From Equations (8) and (9), the unit ramp response c(t) can be written as,

(10) $$c(t)=\frac{(t-\frac{1}{a})+\frac{1}{a}\cdot e^{-at}}{\tau_{d1}\cdot a},t\geq 0$$

From Equation (10), the steady-state error $\varepsilon_{\infty }$ and the response time *t_r_*, the time when the response value reaches 0.95 of the steady-state, can be expressed as,

(11) $$\varepsilon_{\infty }=\frac{1}{\tau_{d1}\cdot a^{2}}$$

(12) $$t_{r}=\frac{3}{a}$$

From Equations (11) and (12), it is obvious that the time constant is proportional to steady-state error and response time.

Secondly, as seen from Equation (4), it is shown that the test statistic includes both the ionospheric gradient and high frequency noise. In order to more accurately detect the change of the ionospheric gradient, the linear time-invariant low-pass filter is adopted by traditional CCD methods to suppress the effect of high frequency noise on the test statistic. Therefore, the relationship between the time constant and estimation accuracy is discussed in the frequency domain.

We know that the cut-off frequency of the system can reflect the ability of denoising. From Equation (8), we can get the relationship between the cut-off frequency of system *w_b_* and the time constant *τ_d_*_1_,

(13) $$w_{b}=a$$

where *w_b_* is defined as the frequency when the amplitude of the amplitude-frequency characteristic curve descends by −3 dB. a is in inverse proportion to *τ_d_*_1_. We find that the time constant is in inverse proportion to estimation accuracy.

Finally, from Equations (12) and (13), we find that there is a counterbalance between the estimation accuracy of the ionospheric gradient and the response time to the ionospheric gradient anomaly, regardless of the time constant.

Regarding the CCD-1OF method, the unit ramp response $c_{1}(t)$, response time $t_{1r}$ and cut-off frequency *w_b_*_1_ of the CCD-2OF method can be expressed as:

(14) $$c_{1}(t)=\frac{t-\frac{2}{a}-\frac{2}{a}(1+a\cdot t)\cdot e^{-at}}{\tau_{d1}^{2}\cdot a},t\geq 0$$

(15) $$t_{1r}=\frac{4.1}{a}$$

(16) $$w_{1b}=\sqrt{\sqrt{2}-1}\cdot a$$

Assuming that the sample time is constant, and *τ_d_*_1_ = *τ_d_*_2_, the time constant is different from the CCD-1OF. From Equations (15) and (16), we find that the time constant is in direct proportion to response time and in inverse proportion to estimation accuracy. Therefore, there is a common shortcoming between CCD-1OF and CCD-2OF with the fixed time constant.

In summary, the above analysis shows that the estimation accuracy of the ionospheric gradient and response time to the ionospheric gradient anomaly of the traditional CCD methods cannot be improved simultaneously by just adjusting the time constants. In order to improve the estimation accuracy and response time at the same time, the test statistic must be constructed in a new method.

## 3. A Two-Step CCD Monitor Approach

It is clear that the Kalman filter method is an optimal way to detect in real time the change of the ionospheric gradient, but the real-time estimation accuracy depends on the knowledge of the noise statistics and the system model. Because of the difficulty in obtaining a precise prior measurement noise characteristic and the much bigger high frequency noise in Equation (4), the real-time estimation accuracy may be low or even divergent. Therefore, we adopt a linear time-invariant low-pass method first to provide a prior measurement noise to describe the statistical characteristic of the measurement noise and to suppress high frequency noise in Equation (4). Then, a system model is designed based on the ionospheric gradient and its change rate, which can describe the small change of the ionospheric gradient. In this way, the new test statistic is designed by a TSA to monitor the CCD anomaly, with the threshold mentioned in Equation (7).

### 3.1. The First Step

Since the cascaded first-order linear time-invariant low-pass filter model has better performance on both estimation accuracy and response time to anomaly than the single first-order linear time-invariant low-pass filter model, we adopt the former model as the first step of TSA,

(17) $$temp_{k}=\frac{\tau_{TSA1}-T_{s}}{\tau_{TSA1}}temp_{k-1}+\frac{1}{\tau_{TSA1}}\left(z_{k}-z_{k-1}\right)$$

(18) $$M_{k}=\frac{\tau_{TSA2}-T_{s}}{\tau_{TSA2}}M_{k}+\frac{1}{\tau_{TSA2}}temp_{k}$$

where *temp_k_* is an intermediate variable, *M_k_* is the output value of the cascaded first-order linear time-invariant low-pass filter and *τ_TSA_*_1_ and *τ_TSA_*_2_ are the time constants of TSA. In order to get the stronger ability of denoising and response time, we assume that *τ_TSA_*_1_ = *τ_TSA_*_2_. Through the analysis of the relationship between response time and time constant in Section 2.2, in order to more quickly and accurately track the ionospheric gradient, we must choose the smaller time constants in the first step; the decision about the time constants is explained in Section 4. By the cascaded first-order linear time-invariant low-pass filter, a prior measurement noise is provided with the corresponding time constants, and the high frequency noise is suppressed in the difference between adjacent epoch CMC, that is *M_k_*. Next, an adaptive Kalman filter model is designed to estimate in real time the ionospheric gradient.

### 3.2. The Second Step

We know that the ionospheric gradient slightly changes in time and space [19]. In order to describe the ionospheric gradient accurately, a system model is designed based on the ionospheric gradient and its change rate. Assuming that the change of the ionospheric gradient rate is constant, the ionospheric gradient and the change of the ionospheric gradient rate can be expressed as [15,18]:

(19) $$I_{g,k}=I_{g,k-1}+T_{s}\cdot dI_{g,k-1}$$

(20) $$dI_{g,k}=dI_{g,k-1}$$

where *I_g_* is the ionospheric gradient, *dI_g_* is the change of the ionosphere gradient rate and *T_s_* is the sample time. *X* = *[I_g_ dI_g_]^T^* is the state vector of the system model. Therefore, the state model of the ionospheric gradient is defined as:

(21) $$X_{k}=\Phi_{k,k-1}X_{k-1}+W_{k-1}$$

where ***W*** is the processing noise and ***Φ*** is the transition matrix, which can be written as:

(22) $$\Phi_{k,k-1}=\left[\begin{array}{cc} 1 & T_{s}\\ 0 & 1 \end{array}\right]$$

The measurement equation of the system is written as follows:

(23) $$Z_{k}=H_{k}X_{k}+V_{k}$$

where ***V_k_*** is the measurement noise, which is a Gaussian white noise sequence, ***Z_k_*** is the observation and ***H_k_*** is the measurement matrix. With the use of Equation (4), ***Z_k_*** and ***H_k_*** can be written as:

(24) $$Z_{k}=M_{k},H_{k}=\left[\begin{array}{cc} 2T_{s} & T_{s}^{2} \end{array}\right]$$

In order to estimate the ionospheric gradient accurately, an adaptive Kalman filter by adjusting the variance-covariance matrix of process noise ***Q*** is adopted to describe the system model accurately for the change of the ionospheric gradient rate, because the change of the ionospheric gradient rate is not observed, and the prior measurement noise is provided in the first step.

The difference between the current measurement and *a priori* estimate ${\tilde{Z}}_{k}$ is written as:

(25) $${\tilde{Z}}_{k}=Z_{k}-{\hat{Z}}_{k,k-1}=Z_{k}-H_{k}\Phi_{k,k-1}{\hat{X}}_{k-1}$$

where the change of the ionospheric gradient is introduced in ${\tilde{Z}}_{k}$. Therefore, for non-stationarity, ***Q*** can be estimated as [26,27]:

(26) $${\hat{Q}}_{k}=K_{k}{\tilde{Z}}_{k}{\tilde{Z}}_{k}^{T}K_{k}^{T}$$

where ***K*** is the gain of the system. The contribution of ${\tilde{Z}}_{k}^{}$ to estimation depends on ***K***.

Finally, the adaptive Kalman filter by adjusting ***Q*** to estimate the ionospheric gradient can be written as follows:

(27) $$\{\begin{array}{c} {\hat{X}}_{k,k-1}=\Phi_{k,k-1}{\hat{X}}_{k-1}\\ P_{k,k-1}=\Phi_{k,k-1}P_{k-1}\Phi_{k,k-1}^{T}+{\hat{Q}}_{k-1}\\ K_{k}=P_{k,k-1}H_{k}^{T}{\left(H_{k}P_{k,k-1}H_{k}^{T}+R_{k}\right)}^{-1}\\ P_{k}=(I-K_{k}H_{k})P_{k,k-1}\\ {\hat{X}}_{k}={\hat{X}}_{k,k-1}+K_{k}(Z_{k}-H_{k}{\hat{X}}_{k,k-1})\\ {\tilde{Z}}_{k}=Z_{k}-H_{k}\Phi_{k,k-1}{\hat{X}}_{k-1}\\ {\hat{Q}}_{k}=K_{k}{\tilde{Z}}_{k}{\tilde{Z}}_{k}^{T}K_{k}^{T} \end{array}$$

where the initial conditions are given as follows,

(28) $$\left\{ \begin{array}{l} X_{0}=\left[\begin{array}{c} 0\\ 0 \end{array}\right]\\ Q_{0}=E(W_{0}W_{0}^{T})=\left[\begin{array}{cc} Q_{I_{g}} & 0\\ 0 & Q_{dI_{g}} \end{array}\right]\\ Z_{1}=M_{1}\\ R_{1}=E(V_{1}V_{1}^{T})=Q_{I_{g}} \end{array}\right.$$

where ***Q_Ig_*** represents the variance calculated by the first step of TSA with the corresponding time constant in the normal condition; we assume that ***Q_Ig_*** = ***Q_dIg_***, *M*_1_ is the first advanced measure in the first step of TSA.

**Figure 2:**
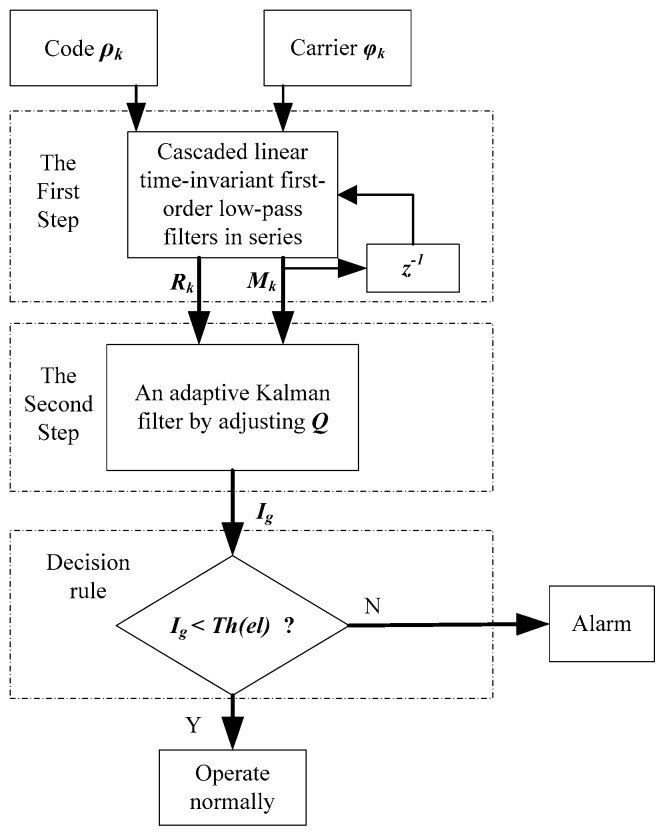
Schematic of proposed TSA ionospheric gradient detection.

As discussed above, we know that TSA has the advantages of estimation accuracy and response time to anomaly. Therefore, the new test statistic is adopted by TSA to monitor the ionospheric gradient anomaly. Firstly, the cascaded first-order linear time-invariant low-pass filter model is used to construct a new measurement ***M_k_*** with a shorter time constant to provide the measurements, and the prior measurement noise ***R_k_*** is provided with the corresponding time constant. Secondly, an adaptive Kalman approach by adjusting the covariance ***Q*** is used to estimate ionospheric gradient *I_g_* in real time. Finally, the ionospheric gradient of the estimation is regarded as the test statistic for TSA. The detection threshold is designed from Equation (7) in Section 2.2. When the test statistic exceeds the detection threshold, an alarm will be raised; otherwise, the system can be supposed to operate normally. The performance of the TSA and the traditional CCD methods is compared in the next section. The processing of a two-step CCD monitor approach is shown in Figure 2.

The benefits of TSA on monitoring the CCD anomaly can be three-fold. Firstly, in the first step of TSA, the cascaded first-order linear time-invariant low-pass filters can provide more accurate *a priori* measurement noise and measurements with the corresponding time constant for the Kalman filter, so that the change of the ionospheric gradient can be detected quickly by the estimation. Secondly, the system model is designed based on the ionospheric gradient and its change rate, which more precisely characterizes the ionospheric variation. Finally, an adaptive Kalman filter by adjusting the covariance ***Q*** is adopted to speed up the response time to the ionospheric gradient anomaly. Superior performance can therefore be anticipated from the proposed TSA algorithm and verified in the experiment analysis.

## 4. Experiment Analysis

In order to reliably evaluate the proposed algorithm, the datasets must reflect typical navigation environments. According to the MOPS requirements on CCD, the simulation data were first carried out to create the respective ionospheric gradient-free and ionospheric gradient anomaly cases, with different standard deviations for noise. Then, the real-world GPS data experiment was conducted to make a comparison between the proposed TSA and the traditional CCD methods in the respective ionospheric gradient-free and ionospheric gradient anomaly cases.

### 4.1. Numerical Simulation

**Figure 3:**
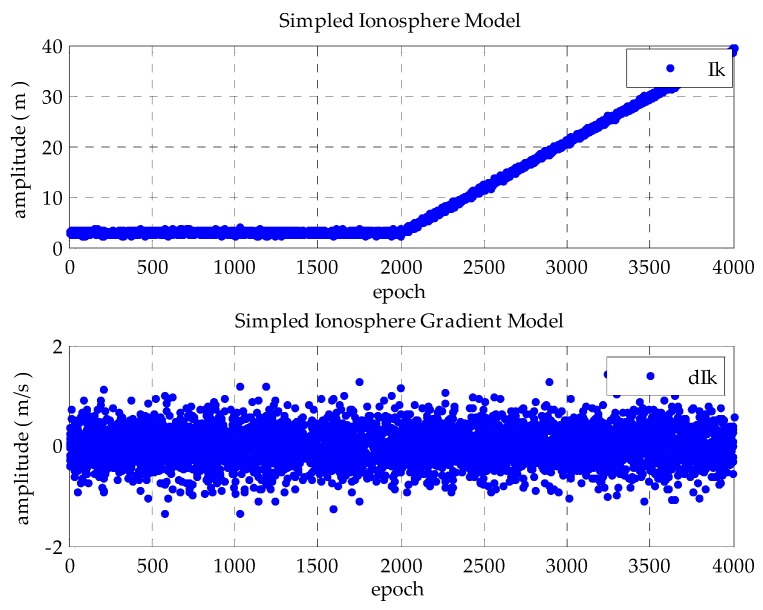
Simulation of the sample ionospheric and ionospheric gradient model in the respective ionospheric gradient-free and ionospheric gradient anomaly cases.

To demonstrate the TSA by numerical simulation, 4000 samples are generated by simulation, and a steep gradient change at a rate of 0.018 occurs after 2000 epochs to simulate the ionospheric anomaly, as *I_k_*. Therefore, a steep gradient is generated by the difference of adjacent samples as *dI_k_* to simulate the respective ionospheric gradient-free and ionospheric gradient anomaly cases. *I_k_* and *dI_k_* are written as:

(29) $$I_{k}=\{\begin{array}{c} 3+n_{k},0<k\leq 2000\\ 3+0.018(k-2000)+n_{k},2000<k\leq 4000 \end{array}$$

(30) $$dI_{k}=\{\begin{array}{c} 0,k=1\\ I_{k}-I_{k-1},k\neq 1 \end{array}$$

where n represents the white Gaussian noise and k represents the k-th epoch. When *n* ~ *N* (0, 0.25), the simple ionosphere model and simple ionospheric gradient model are shown in Figure 3, where the first 2000 epochs are ionospheric gradient-free cases; the others are ionospheric gradient anomaly cases.

**Figure 4:**
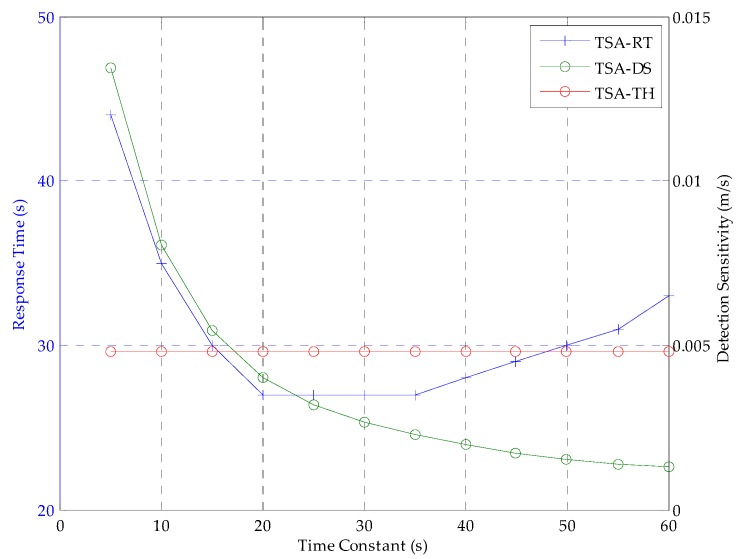
Relationship between time constant and response time to anomaly, detection sensitivity when *n_k_* ~ *N* (0, 0.25).

In order to detect the ionospheric gradient at a rate of 0.018, the decision about the time constant is very important in the first step of TSA. Taking both detection sensitivity and response time into consideration, the time constant is chosen when the performance of TSA is superior to CCD-2OF, with the recommended time constants. Therefore, 20 s is the better choice as the time constant of TSA when *n_k_* ~ *N* (0, 0.25), as shown in Figure 4, where: the green line is the detection sensitivity (DS) of TSA with corresponding time constants; the red line is the threshold value (TH) of the detection of the sensitivity of TSA decided by CCD-2OF with 30 s; the blue line represents the response time (RT) to the anomaly of TSA. When the standard deviation of *n_k_* is changed, the approximate time constant will be chosen from 100 Monte Carlo simulations. The time constants of 20 s, 30 s, 45 s, 50 s and 55 s are mapped to the noise with standard deviations of 0.25, 0.5, 1.5 and 2, respectively. This shows that the larger the standard deviation of noise is, the larger the time constant of TSA is chosen.

**Figure 5:**
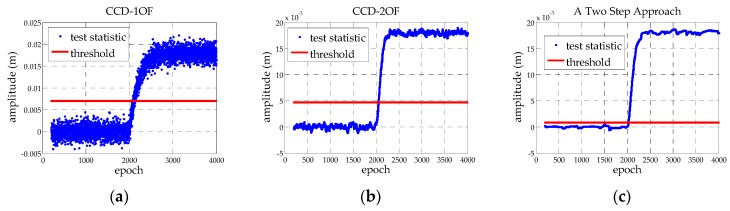
(**a**–**c**) Sample anomaly detection by CCD-1OF, CCD-2OF and TSA, respectively.

To compare the simulated gradient detection performance, the test statistics are constructed by CCD-1OF using *τ_d_*_1_ = 200 s from Equation (5), CCD-2OF using *τ_d_*_1_ = *τ_d_*_2_ = 30 s from Equations (5) and (6) [5] and the TSA using *τ_TSA_*_1_ = *τ_TSA_*_2_ = 20 s, when *n_k_* ~ *N* (0, 0.25), shown in Table 1. The thresholds for the three methods are determined by Equation (7), where *K_ffd_* is chosen from the required probability of false alarms [28]. The inflation factor f is determined as 1, because the noise obeys a Gaussian distribution. In order to obtain the thresholds in normal conditions, which are mentioned in MOPS, the thresholds are calculated from the 200th sample, ending with the 2000th sample. Figure 5 represent the test statistics and the thresholds of CCD-1OF, CCD-2OF and TSA, respectively. Through the comparison of the three methods, the response time to anomaly, detection sensitivity and the standard deviation are: 84, 32, 28; 0.0071, 0.0047, 0.0023; and 0.0012237, 0.00081983, 0.00041461, respectively. The results show that the TSA has higher detection sensitivity and a faster response time to anomaly.

When the standard deviation of *n_k_* is changed, the average response time and detection threshold are shown in Table 1 and Table 2 by the three methods above and the advanced CCD-2OF (ACCD-2OF), which has the same time constant as TSA from 100 Monte Carlo simulations, respectively. From Table 1 and Table 2, we find that the TSA has a quicker response time to anomaly and a higher detection sensitivity than the traditional CCD methods. Compared to ACCD-2OF, it proves that the second step of TSA further improves the response time to the anomaly and detection sensitivity. N means the monitor approach that is adopted fails to detect the anomaly, in Table 1.

As discussed above, it is necessary to adjust the time constant based on the noise, because the TSA and ACCD-2OF can detect samples anomaly with corresponding time constant, even when the noise is larger, and the optimal time constant can improve the CCD methods’ performance. Additionally, depending on the *a priori* information from the first step, the performances of the detection sensitivity and response time to anomaly are further improved by the second step of TSA. The numerical comparisons with traditional CCD monitoring methods under the ionospheric gradient-free and ionospheric gradient anomaly cases demonstrate that the TSA has a quicker response time to anomaly and a higher detection sensitivity with the corresponding time constant.

### 4.2. Real Data Simulation

To further assess the proposed ionospheric anomaly monitoring approach, which is superior to traditional CCD methods on both response time to ionospheric gradient anomaly and the estimation accuracy of ionospheric gradient, the real-world GPS data are collected at Beijing University of Civil Engineering and Architecture. The proposed approach is tested on 18 h 30 min data at a 1-Hz sampling time on 22 November 2013.

**Figure 6:**
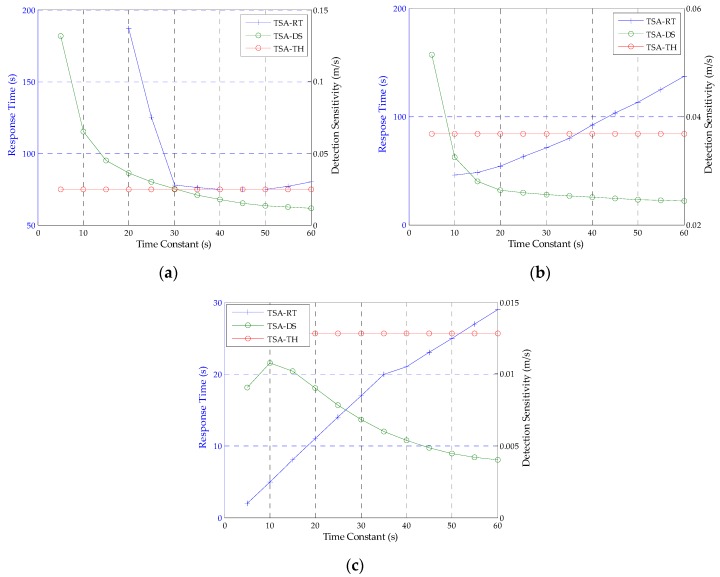
(**a**–**c**) The relationship of TSA between the time constant and response time, detection sensitivity, when 0 < *ele* ≤ 30, 30 < *ele* ≤ 50, 50 < *ele* ≤ 90, respectively.

We know that measurement noise is different at different elevations because multipath noise is related to elevation. Therefore, we must decide the time constants of TSA at different elevations. First of all, the raw measurements of each satellite are divided into 9 groups according to the elevation; each group contains 10 degrees. Secondly, each satellite has the ionospheric gradient changing at a rate of 0.018 m/s in each group. Finally, in order to get a quicker response time to the ionospheric gradient anomaly than traditional CCD methods, the range of the time constant of TSA is between 5 and 30 s. The optimal time constants should obtain a balance between fast response time and high detection sensitivity. These factors are related to the effect of measurement noise. The choice criterion of time constants, inspired by [5], in the first step of TSA is that the detection sensitivity of TSA is better than CCD-2OF, and the response time to anomaly of TSA is as quick as possible. The response time and detection sensitivity performance based on different time constants are also given based on simulations for each ten degrees. With a test among 31 satellites, we have found that the optimal time constants can be observed at the selected categorized elevation groups, *i.e.*, 0 < *ele* ≤ 30, 30 < *ele* ≤ 50, 50 < *ele* ≤ 90. Thus, we determine the time constants as shown in Table 3. It shows that the higher the elevation group is, the smaller is the time constant of TSA chosen. Figure 6 shows the optimal time constants chosen at the selected categorized elevation groups for pseudo-random noise code (PRN) 19. In Figure 6a, we find that the response time decreases, while the response time increases in Figure 6c when the time constant grows. This is because of the variation of measurement noise at different elevations. Since the response time of TSA is determined by the cascaded first-order low-pass filters in the first step and the Kalman filter in the second step, regarding Figure 6a, with severer noise at low elevations, the Kalman filter needs a long time to mitigate the effect of noise with smaller time constants in the first step, which would cause the longer response time. Similarly, the effect of noise is relatively smaller at high elevations, such as Figure 6c, although, the Kalman filter needs a relatively shorter time to mitigate the effect of noise; the larger time constants in the first step would also cause a longer response time.

**Figure 7:**
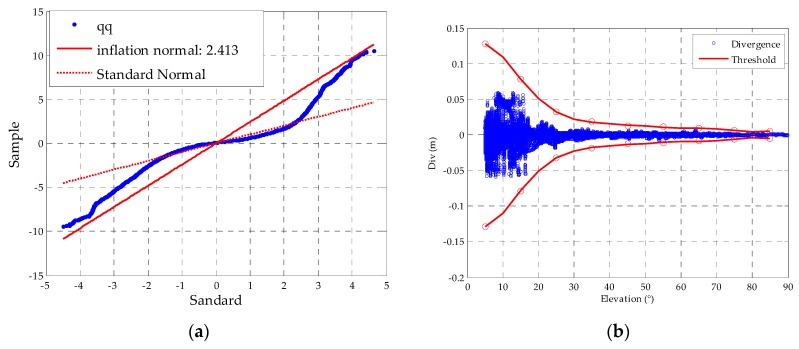
(**a**) Quantile-quantile plot for the inflation factor; (**b**) distribution of the test statistics with respect to satellite elevations and the resultant thresholds.

**Figure 8:**
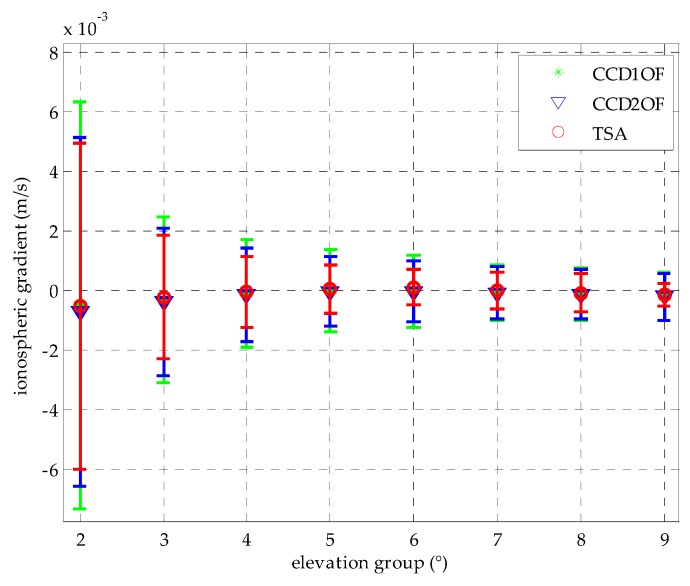
Comparison of the mean and deviation of the estimation between CCD-1OF, CCD-2OF and TSA.

After the time constant is chosen in each elevation group, the detection thresholds in terms of satellite elevations at intervals of 10° are computed using the mean and standard deviation of the TSA in normal conditions. In addition, *K_ffd_* is set to 5.73 again, and the inflation factor f is chosen from Figure 7a. Figure 7b shows that test statistics and the determined thresholds with respect to the elevation angle at each bin; we interpolate the thresholds at each bin with the 7th polynomial fit as the red circle. Figure 8 shows that the mean and the standard deviation distribution comparing between TSA and the traditional CCD methods with the corresponding time constant. The results show that the standard deviation of TSA is better than CCD-1OF and CCD-2OF under normal conditions. Because the standard deviation is much larger in the first group, we just show the other groups in Figure 8. The average estimation accuracy of TSA compared to the CCD-1OF and CCD-2OF improves 41.3% and 31.3%, respectively. From Figure 8, it is shown that the standard deviation decreases with the increasing of elevation angles for each CCD method.

**Figure 9:**
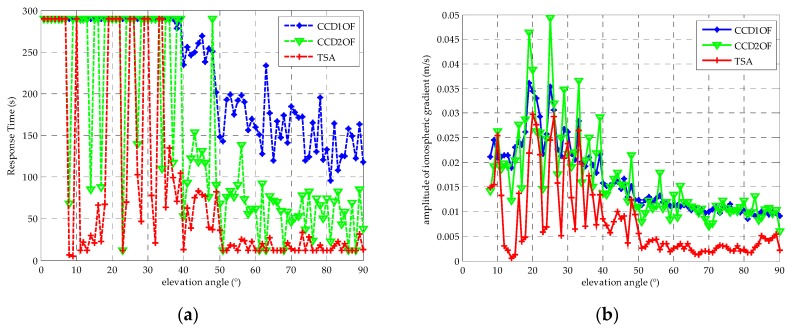
(**a**) The comparison of the response time to anomaly between TSA and the traditional CCD methods with the change of ionospheric gradient rate being 0.018 m/s for PRN 19; (**b**) the comparison of the detection sensitivity between TSA and the traditional CCD for PRN 19.

Next, to simulate the impact of a severe ionospheric gradient, the ionospheric gradient at a rate of 0.018 m/s is inserted into the normal code minus the phase in the adjacent epoch at every single degree elevation angle for each satellite for a duration of 290 s in PRN 19. The satellite of Prn19 is chosen in simulation, because it traverses elevation as much as possible. The time constant of TSA is chosen from Table 3. Figure 9a shows the response time in each elevation bin between TSA and the traditional CCD methods for the PRN19 satellite. Table 4 shows the mean and standard derivation of response time to anomaly between TSA and the traditional CCD methods from Figure 9a. The result shows that TSA has a lower mean and standard deviation of response time than CCD-1OF and CCD-2OF, namely the TSA has a quicker response time to the ionospheric gradient anomaly.

Furthermore, the detection sensitivity is compared between TSA and the traditional CCD methods for PRN19 in Figure 9b. The result shows that the TSA has higher detection sensitivity than the traditional CCD methods. In general, with the increasing of elevation angle, we will get a smaller detection threshold. Once the ionospheric gradient is smaller than the detection threshold of the corresponding elevation bin, we cannot detect the anomaly, as shown in Figure 9a.

**Figure 10:**
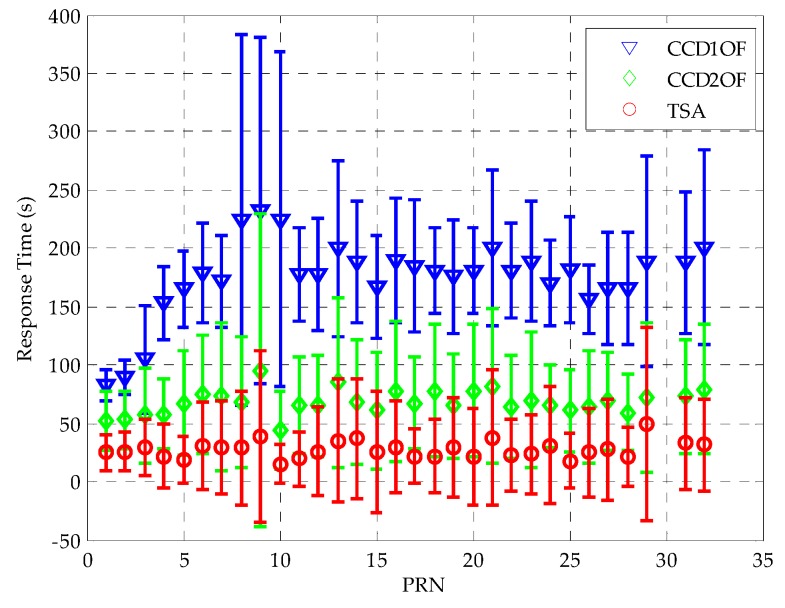
Comparison of the overall average response time to anomaly with respect to PRN.

Finally, we compare the mean and standard deviation of the response time to anomaly with respect to all of the satellites visible when the ionospheric gradient is at a rate of 0.018 m/s in each single elevation angle. The result shows that TSA has a quicker response to detect the CCD anomaly for all satellites visibly, as shown in Figure 10. The average response time to anomaly of TSA compared to CCD-1OF and CCD-2OF improves by 83.7% and 59.3%, respectively.

## 5. Conclusions

Ionospheric anomaly monitoring is an important integrity monitor procedure for GBAS. The contribution of this paper is to develop and test a new ionospheric anomaly monitoring algorithm by using the GPS receiver, which overcomes the traditional CCD monitoring methods that the estimation accuracy and response time to anomaly cannot be improved simultaneously with a fixed time constant for GBAS, achieving the real-time detection of the first-order ionospheric gradient. From our simulations, it is shown that the TSA provides a better ionospheric gradient monitoring performance, which is reflected by the response time to anomaly and the detection sensitivity, compared to the traditional CCD methods, such as CCD-1OF and CCD-2OF. The estimation accuracy of the ionospheric gradient, response time and detection sensitivity to the ionospheric gradient anomaly results from the real-world GPS data has also indicated that the TSA provides a better ionospheric gradient monitoring performance. By deriving the test statistic *Ig* based on integrating the cascaded linear time-invariant low-pass filters with the adaptive Kalman filter, the TSA achieves the superior ionospheric gradient monitoring performance using two innovations: (1) the TSA adopts an optimal time constant in the first step with an *a priori* noise characteristic, unlike the traditional CCD methods with fixed time constants; (2) the Kalman system model is designed based on the ionospheric gradient and its change rate by adjusting the variance-covariance matrix ***Q***, which more precisely characterizes the ionospheric variation.

Note that these results are based on limited data; we are conducting further work to fully characterize the performance of the TSA under different operational conditions, especially the determination of the time constant at different elevations. Through the research of this paper, the future research work includes: first, we regard the multipath and receiver noise as the Gaussian white noise in the TSA; therefore, the new Kalman model should be constructed to overcome the assumption; second, the *a priori* information of TSA is greater than the traditional CCD methods; therefore, the adaptive time constant model should be constructed to reduce the amount of calculation.

## Figures and Tables

**Table 1:** Comparing the response time to anomaly of four methods in different standard deviations of noise. ACCD, advanced CCD.

Standard Deviation of *n_k_*	Average Response Time (epoch)			
	CCD-1OF (*τ_d_*_1_ = 100)	CCD-2OF (*τ_d_*_1_ = *τ_d_*_1_ = 30)	ACCD-2OF (*τ_d_*_1_ = *τ_d_*_1_ = *τ_TSA_*_1_ = *τ_TSA_*_2_)	TSA (*τ_TSA_*_1_ = *τ_TSA_*_2_)
0.25	72	30	30	28
0.5	159	51	50	42
1	488	104	80	62
1.5	N	324	110	87
2	N	692	140	115

**Table 2:** Comparing the detection sensitivity of four methods in different standard deviations of noise.

Standard Deviation of *n_k_*	Average Detection Threshold			
	CCD-1OF (*τ_d_*_1_ = 100)	CCD-2OF (*τ_d_*_1_ = *τ_d_*_1_ = 30)	ACCD-2OF (*τ_d_*_1_ = *τ_d_*_1_ = *τ_TSA_*_1_ = *τ_TSA_*_2_)	TSA (*τ_TSA_*_1_ = *τ_TSA_*_2_)
0.25	0.0072	0.0044	0.0018	0.0011
0.5	0.0144	0.0089	0.0035	0.0021
1	0.0287	0.0179	0.0071	0.0044
1.5	0.0431	0.0265	0.0106	0.0068
2	0.0574	0.0354	0.0142	0.0091

**Table 3:** Time constant of TSA for different elevation groups chosen depending on 31 satellites being visible.

Elevation Range (°)	*ele* ≤ 30	30 < *ele* ≤ 50	50 < *ele* ≤ 90
Time constant (s)	$\tau_{TSA1}=\tau_{TSA2}=30$	$\tau_{TSA1}=\tau_{TSA2}=20$	$\tau_{TSA1}=\tau_{TSA2}=10$

**Table 4:** Mean and standard deviation of the response time between TSA and the traditional CCD methods with the change of ionospheric gradient rate being 0.018 m/s for PRN19.

Method	CCD-1OF (*τ_d_*_1_ = 100 s)	CCD-2OF (*τ_d_*_1_ = *τ_d_*_1_ = 30 s)	TSA (*τ_TSA_*_1_ = *τ_TSA_*_2_)
Mean (s)	176	65	30
Standard Deviation (s)	48.45	44.27	42.74
